# Cetuximab potentiates oxaliplatin cytotoxic effect through a defect in NER and DNA replication initiation

**DOI:** 10.1038/sj.bjc.6604134

**Published:** 2008-01-08

**Authors:** D Balin-Gauthier, J-P Delord, M-J Pillaire, P Rochaix, J-S Hoffman, R Bugat, C Cazaux, P Canal, B C Allal

**Affiliations:** 1 EA 3035 Laboratoire de Pharmacologie Clinique et Expérimentale des Médicaments Anticancéreux, Université Paul Sabatier, Toulouse, France; 2 Institut Claudius Regaud, 20-24, rue du Pont Saint Pierre, Toulouse Cedex 31052, France; 3 équipe Instabilité génétique et cancer du département ‘Mécanismes de surveillance du génome’ Institut de Pharmacologie et de Biologie Structurale, CNRS UMR 5089 (IPBS) 205 route de Narbonne, Toulouse Cedex 31077, France

**Keywords:** cetuximab (Erbitux®, C225), oxaliplatin, mechanisms, NER, replication

## Abstract

Preclinical studies have demonstrated that the chemotherapeutic action of oxaliplatin, a third generation platinum derivative, is improved when combined with cetuximab, a monoclonal antibody inhibitor of epidermal growth factor receptors. To explore the mechanism of this synergistic benefit, we used HCT-8 and HCT-116, two human colon cancer cell lines, respectively, responsive and non-responsive to the oxaliplatin/cetuximab combination. We examined the effect of drug exposure on glutathione-*S*-transferase-mediated oxaliplatin detoxification, DNA–platinum adducts formation, cell cycle distribution, apoptosis, and the expression of multiple targets involved in DNA replication, recombination, and repair. The major changes we found in HCT-8 were a stimulation of oxaliplatin–DNA adduct formation associated with reduced expression of the key enzyme (excision repair cross complementation group1: *ERCC1*) in the key repair process of oxaliplatin–DNA platinum adduct, the nucleotide excision repair (NER), both at the mRNA and protein levels. We also observed a reduced expression of factors involved in DNA replication initiation, which correlates with an enrichment of cells in the G1 phase of the cell cycle as well as an acceleration of apoptosis. None of these changes occurred in the non-responsive HCT-116 cell that we used as a negative control. These findings support the fact that cetuximab potentiates the oxaliplatin-mediated cytotoxic effect as the result of inhibition of NER and also DNA replication initiation.

Colorectal cancer is the second leading cause of cancer-related death in the western world. Even though progress has been made in adjuvant chemotherapy, more than 50% of patients relapse or present with metastatic disease at the time of diagnosis.

Oxaliplatin, a third-generation platinum analogue, showed significant single-agent activity in advanced colorectal cancer. Oxaliplatin-mediated cytotoxic effects result on DNA damages via DNA platinum adducts formation. Despite recent improvement in the field of systemic chemotherapy, one of the greatest issues remains is drug resistance. Mechanisms of resistance to platinum agents mainly involve a potentiation of the glutathione-*S*-transferase (GST) detoxification system and/or DNA repair. Elevated levels of GST have been demonstrated to render some cancer cell types resistant against well-known platinum anticancer drugs (Blyumenberg *et al*, 1996; Wernyj and Morin, 2004). Indeed, platinum complexes are very reactive with the cysteine residue of glutathione (GSH), which detoxifies these compounds by a rapid binding mechanism. The nucleotide excision repair (NER) is the main cellular process involved in the elimination of oxaliplatin–DNA platinum adducts (Chaney *et al*, 2005) where excision repair cross complementation group1 (ERCC1) is the key enzyme leading to the 5′ incision of the platinum DNA damage (Reed, 1998). Excision repair cross complementation group1 exists as mRNA and as protein in at least two forms. One form is the full length and the second form is an alternatively spliced form (Yu *et al*, 1998).

Novel biological agents, such as cetuximab (C225, Erbitux®), a recombinant human/mouse monoclonal antibody against epidermal growth factor receptors (EGFR), have recently been shown to improve the clinical benefit for patients with metastatic colorectal cancer (Cunningham *et al*, 2004; Saltz *et al*, 2004). Epidermal growth factor receptors is a member of HER tyrosine kinase growth factor receptor family, involved in signalling pathways affecting cellular growth, differentiation and proliferation (Carpenter and Cohen, 1990). Colorectal cancer is frequently associated with high expression levels of EGFR (25–80%) resulting in a more aggressive disease and a poor prognosis (Porebska *et al*, 2000; Lee *et al*, 2002). Moreover, it has been shown that cetuximab use can partially reverse chemoresistance occurring during irinotecan chemotherapy (Prewett *et al*, 2002).

It is now well documented that EGFR inhibition is involved in resistance to cytotoxic drugs. Hence, the blockade of EGFR is a promising strategy to improve colorectal cancer treatment.

We previously demonstrated on a panel of human colorectal cancer cell lines that cetuximab can enhance the antitumour activity of oxaliplatin and the observed responses were strictly dependent on the cell type, not correlated with the level of EGFR expression but related to the basal level of phospho-EGFR. In this previous work, we have shown that oxaliplatin combined with cetuximab was synergistic in the HCT-8 cell line *in vitro* for inhibition of cellular proliferation and *in vivo* for inhibition of tumour growth, whereas the HCT-116 cell line remained unresponsive (Balin-Gauthier *et al*, 2006).

However, the mechanisms responsible for the combined effects of cetuximab with oxaliplatin remain unclear, as do the mechanisms involved in the reversion of drug resistance.

The aim of this study was to decipher the molecular basis of how cetuximab enhances oxaliplatin's antiproliferative effects; basically, how EGFR signalling pathway inhibition regulates oxaliplatin pharmacology. To explore the mechanism of this synergistic benefit, we used responsive (HCT-8) and non-responsive (HCT-116) human colon cancer cell lines and we analysed the effect of cetuximab treatment on oxaliplatin intracellular detoxification, DNA adduct formation, cell cycle distribution, and induction of apoptosis. We also performed a multigene analysis, by RT-qPCR, to analyse the effect of additional cetuximab treatment on the transcriptional expression of numerous genes involved in cell cycle progression and DNA replication and repair. The results indicate that cetuximab potentiates the oxaliplatin-mediated cytotoxic effect through combined defects in NER and DNA replication initiation.

## MATERIALS AND METHODS

### Drugs

Cetuximab (Erbitux, C225, 2 mg ml^−1^) was kindly provided by MERCK (Darmstadt, Germany) and oxaliplatin (Eloxatine®, L-OHP, 5 mg ml^−1^) was purchased from Sanofi-Aventis (Gentilly, France).

### Cell lines

Human colon carcinoma cell lines HCT-116 and HCT-8 (American Type Culture Collection, ATCC-LGC Promochem Sarl, Molsheim, France) were routinely maintained in RPMI-1640 medium containing 5% fetal calf serum (FCS), supplemented with 2 mM L-glutamine (Cambrex, Saint Beauzire, France) at 37°C and under 5% CO_2_. Before any experiment, cells were cultured for 1 week in phenol-red-free medium complemented as described previously.

### Platinum adduct quantitation

#### Oxaliplatin–DNA adduct formation

Exponentially growing cells were treated with 5 *μ*M oxaliplatin with or without cetuximab (20 *μ*g ml^−1^) for 24 h. Cells were then washed twice with cold PBS and frozen at −20°C until analysis. DNA was extracted and DNA platinum content analysis performed by atomic absorption spectroscopy (AAS) as described below.

#### Oxaliplatin–DNA adduct repair

Exponentially growing cells were treated for 1 h with 40 *μ*M oxaliplatin with or without cetuximab (20 *μ*g ml^−1^). Following incubation, the cells were washed twice with cold PBS and either frozen at −20°C (T0) or placed in a culture medium for 6 and 24 h with 20 *μ*g ml^−1^ added cetuximab for the combination treatment. For the 24-h time points, the cells were incubated with 2.5 *μ*Ci [^3^H]-thymidine 24 h before oxaliplatin exposure to correct for DNA adduct concentrations from replication. DNA was extracted and DNA platinum content analysis performed by AAS as described below.

#### Platinum adduct analysis

DNA from frozen samples was extracted using the Flexigene Kit (Qiagen, Courtaboeuf, France) following the manufacturer's instructions. The samples were stored at −20°C and sonicated just before analysis.

Platinum (Pt) content was determined by AAS on an AAnalyst 600 (Perkin-Elmer, Courtaboeuf, France). A total of 20 *μ*l of DNA solution was pipetted and the temperature increments were 120°C for 20 s, 130°C for 50 s, 350°C for 20 s, 550°C for 20 s, and 1400°C for 20 s. These steps were repeated four times and were followed by further temperature increases to 2350°C for 8 s, 2450°C for 4 s, 20°C for 10 s, and 2450°C for 4 s. The reading wavelength was set to 265.9 nm. Pt concentrations in the samples were calculated from a standard curve (5.25–262.5 ng Pt per ml). Concentrations determined at 6 and 24 h after the end of incubation were corrected by the ratio of thymidine incorporation between the start of the incubation and the time points. Results (pg Pt per *μ*g DNA) are expressed as the mean±s.e.m. of six independent experiments.

### Glutathione-*S*-transferase activity

The total GST activity was determined according to Habig *et al* (1974). Briefly, 2 × 10^6^ exponentially growing cells were plated in 100-mm Petri dishes and exposed to either oxaliplatin (5 *μ*M), cetuximab (20 *μ*g ml^−1^), or a combination of both. After 6 and 24 h of exposure, the cells were harvested in cold PBS and centrifuged at 1200 **g** for 10 min. Pellets were resuspended in 1 ml PBS and sonicated for 30 s in ice. After centrifugation at 15 000 **g** for 30 min, protein quantitation was performed on supernatants by the Bradford method. The samples were stabilised with 100 *μ*l of 50 mg ml^−1^ bovine serum albumin and then incubated with reduced GSH (2 mM final concentration, Sigma, Saint-Quentin Fallavier, France) for 2 min at 30°C. The reaction was started by adding 1-chloro-2,4-dinitrobenzene (2 mM final concentration). The difference in absorbance at 340 nm during the first 3 min was a linear function of the GST activity. Four absorbance measurements were taken at 30°C every 10 s. Glutathione-*S*-transferase activity in the samples was calculated from a standard curve (0.075–10 U ml^−1^) obtained from serial dilutions of an equine GST stock solution (40 U mg^−1^, Sigma).

Results (*n*=3) are expressed as unit (U) of GST per mg protein and represented as the mean±s.e.m. of variation factors of the GST activity after drug exposure compared to GST activity of untreated cells (variation factor=1).

### Western blotting analysis of ERCC1 expression

Cells were plated at 1.5 × 10^6^ cells in 100-mm Petri dishes, and 24 h later they were exposed to 5 *μ*M oxaliplatin and/or 20 *μ*g ml^−1^ cetuximab for 24 h. Cells were then harvested and lysed in specific buffer (50 mM Tris pH 7.4, 150 mM NaCl, 1% Triton X, 0.1% SDS, 5 mM MgCl_2_, 50 mM NaF, 2 mM PMSF, 10 mM DTT, 2 mM orthovanadate, 5 mg ml^−1^ sodium deoxycholate, 6.4 mg ml^−1^ phosphate substrate, and Sigma 104®). Cleared lysates were separated on 12.5% SDS-polyacrylamide gels, blotted to PVDF membranes (Amersham, Orsay, France), and incubated with specific antibodies against ERCC1 and Tubulin beta (Neomarkers, Interchim, Montlucon, France). Detection was performed using peroxidase-conjugated secondary antibodies (Bio-Rad, Marnes la Coquette, France) and an ECL^+^ chemiluminescence detection kit (Amersham Pharmacia Biotech, Orsay, France). The blots were scanned and analysed with the molecular dynamics densitometer and ImageQuant software. Results are representative of three independent experiments.

### Flow cytometric analysis of cell cycle phase distribution

Exponentially growing cells were plated at 10^5^ cells in 100-mm Petri dishes, and 24 h later the cells were exposed to 5 *μ*M oxaliplatin, 20 *μ*g ml^−1^ cetuximab, or the combined treatment according to three schedules of exposure: (1) 24-h exposure to oxaliplatin followed by replacement of the medium by drug-free medium until analysis; (2) simultaneous oxaliplatin and cetuximab 24-h exposure followed by cetuximab treatment alone (medium and treatment were changed daily until analysis); and (3) cetuximab alone renewed daily until analysis. At the time indicated in the figure legends, media were collected and cells trypsinised and pelleted by low-speed centrifugation (860 **g** for 5 min). Cells were then washed twice in 0.5 ml of cold PBS and fixed in 1.5 ml of ice-cold absolute ethanol for 1 h at 4°C, followed by staining with propidium iodide (1 mg ml^−1^). The DNA content was determined with a FACScalibur flow cytometer (Becton Dickinson, San Jose, CA, USA). Unless otherwise stated, the proportions of cells in G0/G1, S, and G2/M phases of the cell cycle were calculated from their DNA histogram using ModFit software (Becton Dickinson). Results are representative of three independent experiments.

### Fluorescence

Cells were seeded on glass coverslips into six-well plates at a density of 1 × 10^5^ cells per well in RPMI 1640 phenol-red-free medium supplemented with 5% FCS and 2 mM L-glutamine. Twenty-four hours later, cells were exposed to 5 *μ*M oxaliplatin, 20 *μ*g ml^−1^ cetuximab, or the combined treatment according to one of three schedules of exposure: (1) 24-h exposure to oxaliplatin followed by replacement of medium with drug-free medium until analysis; (2) 24-h exposure to simultaneous oxaliplatin and cetuximab followed by cetuximab exposure alone, renewed daily until analysis; or (3) cetuximab alone, renewed daily until analysis. At the indicated time in the figure legends, cells were fixed in paraformaldehyde (3%), and nuclei were visualised by incubation with DAPI (100 *μ*g ml^−1^). Cells were viewed on a Zeiss Axiophot microscope (objective lenses × 63) and pictures taken with a Princeton camera. Results are representative of two independent experiments in duplicate.

### Phospho-AKT quantitation

The concentration of the phosphorylated form of AKT was measured using a Pathscan™ Sandwich ELISA Kit (Cell Signaling Technology, OZYME, Saint Quentin en Yvelines, France) following the supplier's instructions and expressed as the amount of phosphorylated AKT related to the protein content (fmol mg^−1^ of protein). Briefly, 1.5 × 10^6^ cells were plated in 100-mm Petri dishes, and 24 h later, cells were exposed to 5 *μ*M oxaliplatin and/or 20 *μ*g ml^−1^ cetuximab for 24 h. Cells were then harvested in lysis buffer, and the level of phospho-AKT was quantified according to the supplier's protocol. The total protein quantity was determined according to the Bradford method. Results are expressed as pg of phospho-AKT per *μ*g of total protein and represented as the mean±s.e.m. of the variation factor of the phospho-AKT level compared to the phospho-AKT level of untreated cells (variation factor=1) from three independent experiments.

### Real-time PCR

A total of 2 × 10^6^ exponentially growing cells were plated in 100-mm Petri dishes and exposed for 24 h to 5 *μ*M oxaliplatin, 20 *μ*g ml^−1^ cetuximab, or combination of both treatments. Then, cells were washed twice with PBS, and total RNA was isolated using Trizol solution (Invitrogen Life Technologies, Cergy Pontoise, France). The quality and quantitation of each RNA sample were determined by microchannel electrophoresis assay on the Agilent 2100® Bioanalyser using the RNA NanoLab® chip and the RNA 6000 Nano Assay® kit (Agilent Technologies, Massy, France). A total of 500 ng of total RNA was reverse transcribed using the iScript™ cDNA synthesis kit (Bio-Rad) according to the manufacturer's instructions. Then, quantitative RT-PCR was performed on an iCycler apparatus (Bio-Rad) using the SYBR Green Jumpstart® and Taq ReadyMix® (Sigma) following the supplier's protocol. The sequences for the *GAPDH* and *ERCC1* primers were designed with Beacon designer software (Bio-Rad) and purchased from Proligo (Sigma).

Excision repair cross complementation group1 expression analysis: a total of 500 ng of total RNA was reverse transcribed using the iScript cDNA synthesis kit (Bio-Rad) according to the manufacturer's instructions. Quantitative RT-PCR was performed on an iCycler apparatus (Bio-Rad) using the SYBR Green Jumpstart and the Taq ReadyMix (Sigma) following the supplier's protocol. Briefly, the thermal cycling conditions included an initial Taq polymerase activation (95°C for 3 min); the denaturation step (40 cycles at 95°C for 15 s); the annealing and extension steps (60°C for 1 min); and a melting curve analysis (incubation at 95°C for 10 s, then slowly decreased to 20°C). The *GAPDH* and *ERCC1* primers (Proligo, Sigma) were as follows: (i) *ERCC1* primers: *ERCC1* S 5′-GACCACAGGCTCCAGATGG-3′; *ERCC1* AS 5′-ATCCTCGTCGAGGGGTATCAC-3′; (ii) *GAPDH* primers: *GAPDH* S 5′-TGCACCACCAACTGCTTAGC-3′, *GAPDH* AS 5′-GGCATGGACTGTGGTCATGAG-3′. To avoid amplification of contaminating genomic DNA, one of the two primers was designed to be located at the junction between two exons or in a different exon. First, we validated the PCR efficacy and specificity of the primers. Then, we produced a standard curve with cDNA obtained from 0.1 to 20 ng of total RNA to assess PCR efficacy and a melting curve to check potential dimer formation. No dimers were observed, and the calculated efficacy of PCR was between 0.95 and 1 for all tested genes. Results are expressed as the mean±s.e.m. of at least three independent experiments.

DNA replication and DNA repair factor expression analysis: a total of 2 *μ*g of total RNA was reverse transcribed using the High-Capacity cDNA Archive Kit (Applied Biosystems, APPLERA, Courtaboeuf, France) as recommended by the supplier. Amplification of the replication and repair factors was performed by real-time PCR on the Applied Biosystems 7900HT Fast Real-Time PCR System with the TaqMan® Low Density Array screening technology (Applied Biosystems) following the manufacturer's protocol. References of the primers and probes targeting the replication and factors are as follows: *CDC45L* (ABI cat no. Hs00185895_m1), *CDC6* (ABI cat no. Hs00154374_m1), *CDC7* (ABI cat no. Hs00177487_m1), *SLD5* (ABI cat no. Hs00260545_m1), *MCM7* (ABI cat no. Hs00428518_m1), *MCM8* (ABI cat no. Hs00261182_m1), *ASK* (ABI cat no. Hs00272696_m1), *DNA polymerase α* (ABI cat no. polA, Hs00213524_m1), *CHK1* (ABI cat no. Hs00176236_m1), *Claspin* (CLSPN, ABI cat no. Hs00375405_m1), *MCM2* (ABI cat no. Hs00170472_m1), *RAD51* (ABI cat no. Hs00153418_m1), *DNA polymerase-β* (PolB, ABI cat no. Hs00160263_m1), and *mutL homologue 1 (MLH1)* (ABI cat no. Hs00179866_m1). To normalise gene expression between treated and control samples, control genes (18S, *GAPDH, HPRT, YWHAZ*) were also amplified with the same strategy (ABI cat no. Hs9999901_s1, Hs4342376, Hs99999909_m1, Hs00237047_m1, respectively). qBase software (http://medgen.ugent.be/qbase/) was used to test the expression stability of the control genes. The two most stable control genes, *GAPDH* and *HPRT*, were used by qBase to normalise the expression values of all the replication and repair targets.

### Statistical analysis

All results are expressed as mean±s.e.m. Results were analysed using Student's *t*-tests, and *P*<0.05 was accepted as statistically significant.

## RESULTS

### Cetuximab increases and stabilises the level of platinum–DNA adducts in HCT-8 but not in HCT-116 cells

**Figure 1:**
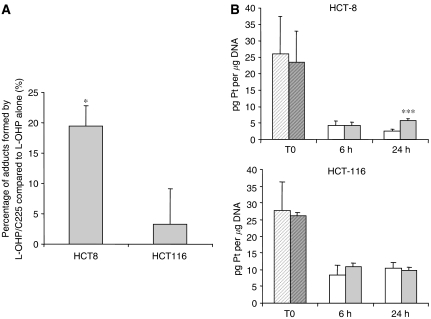
Effect of oxaliplatin combined with cetuximab on platinum–DNA adduct formation and repair. DNA was extracted and platinum content was measured by atomic absorption spectrophotometry. Quantitation of platinum–DNA adducts was performed after (**A**) exposure to either 5 *μ*M oxaliplatin alone or combined with 20 *μ*g ml^−1^ cetuximab for 24 h (*n*=3); (**B**) exposure of the HCT-8 and HCT-116 cell lines for only 1 h (T0) to 40 *μ*M oxaliplatin alone (L-OHP, 
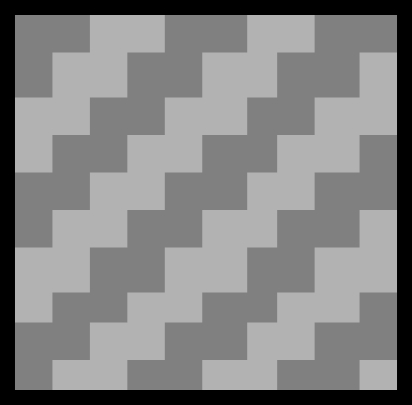
) or combined with cetuximab (C225, 
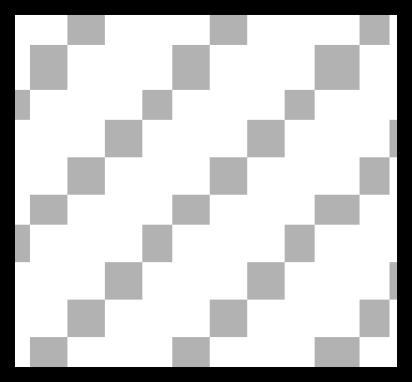
) and followed after medium change by either vehicle (□); or 20 *μ*g ml^−1^ cetuximab (
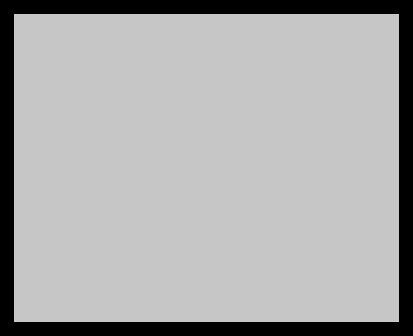
) exposure for 6 and 24 h (*n*=6). Dilution effect of proliferation on the DNA adduct content was corrected by [^3^H]-thymidine incorporation. Values are mean±s.e.m. of at least three independent experiments. ^***^P<0.005; ^*^P<0.05. Student's *t*-test compared to oxaliplatin alone.

The responsive HCT-8 and the non-responsive HCT-116 cell lines were exposed for 24 h to 5 *μ*M oxaliplatin with or without 20 *μ*g ml^−1^ cetuximab, and the DNA platination was then determined by AAS. We found that the level of platinum–DNA adducts in the HCT-8 cells increased by 19.4±3-fold when cetuximab was combined with oxaliplatin (*P*<0.05), whereas no significant differences in the rate of DNA platination were observed for the HCT-116 cell line with the combined treatment (Figure 1A). We then performed a kinetic experiment presented in Figure 1B where we exposed HCT-8 and HCT-116 cells to 40 *μ*M oxaliplatin for only 1 h with or without cetuximab (20 *μ*g ml^−1^) (T0) followed by a subsequent treatment with either vehicle or 20 *μ*g ml^−1^ cetuximab for 6 and 24 h. No difference was observed in either cell line in the platinum–DNA adduct content between oxaliplatin exposure alone and oxaliplatin/cetuximab combination at T0 and *T*=6 h. However, for the HCT-8 cells, the cetuximab treatment resulted in a significant (*P*<0.001) persistence of DNA platination after 24 h (5.75±0.6 pg Pt per *μ*g DNA) compared to the oxaliplatin-alone exposure (2.65±0.5 pg Pt per *μ*g DNA). This outcome was not observed for the non-responsive HCT-116 cell line. These data suggest that cetuximab could compromise the DNA repair process of bulky platinum adducts in the responsive HCT-8 cell line.

### Oxaliplatin and/or cetuximab do not modulate GST activity

One of the cellular pathways involved in the modulation of DNA platination is the detoxification activity of GST, which has a cysteine residue that is very reactive with the platinum complex. Thus, we determined whether cetuximab exposure in cells treated with oxaliplatin enhances GST activity. HCT-8 and HCT-116 cells were exposed 6 and 24 h to cetuximab alone, oxaliplatin alone, or a combination of both, and GST activity was determined as described in Materials and Methods. For both cell lines, we failed to find any difference in GST activity regardless of treatment, demonstrating that the persistence of DNA platination in HCT-8 cells after the combined oxaliplatin/cetuximab treatment did not result from hyperactivity of GST.

### Combination oxaliplatin/cetuximab inhibits oxaliplatin-induced overexpression of ERCC1 through transcriptional regulation in HCT-8

**Figure 2:**
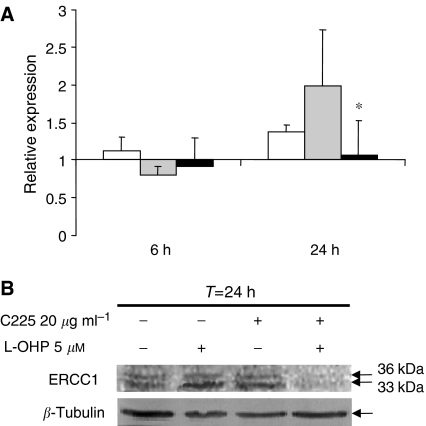
Effect of cetuximab, oxaliplatin, and the combination of both on *ERCC1* expression at the mRNA and protein levels in the HCT-8 cell line. (**A**) Relative quantitation of *ERCC1* mRNA was performed with *GAPDH* as the housekeeping gene and untreated cells as control reference. HCT-8 cells were exposed to 20 *μ*g ml^−1^ cetuximab (C225, □), 5 *μ*M oxaliplatin (L-OHP, 
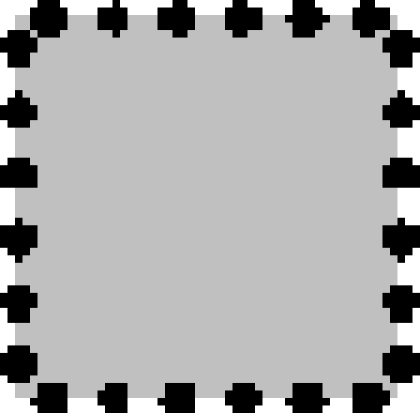
), or a combination of both (▪) for 6 and 24 h. Results are expressed as the mean±s.e.m. of three independent experiments. (**B**) Western blot analysis of ERCC1 protein expression after 24 h exposure to 5 *μ*M oxaliplatin, 20 *μ*g ml^−1^ cetuximab, or a combination of both drugs and representative of three independent experiments. ^*^P<0.05 Student's *t*-test compared to the effect of oxaliplatin alone.

Nucleotide excision repair is based on a cut-and-paste mechanism requiring protein complexes that recognise the DNA damage induced by platinum adduct, with helicases and nucleases that remove the damaged strand, and DNA polymerases and ligases that restore the correct sequence. One critical gene of NER is *ERCC1*, which is involved in the 5′ incision of the damage (Reed, 1998). Quantitative RT-PCR analysis of *ERCC1*, *XPA*, and *XPD* expression were performed in HCT-8 cells that were either untreated, exposed to oxaliplatin alone, or treated with a combination of oxaliplatin/cetuximab (Figure 2A). For this relative quantitation, we first identified the most stable housekeeping gene among *GAPDH*, *HPRT*, and *β*-actin using the geNorm software. Both *β-actin* and *GAPDH* were detected as stable with four cell lines and under experimental conditions. Then normalisation was performed using the ΔΔ*C*_t_ method (Vandesompele *et al*, 2002) with *GAPDH* as the housekeeping gene.

When the cells were exposed to either oxaliplatin or cetuximab alone or to combination of both, neither *XPA* nor *XPD* exhibited major modification (data not shown). On the contrary, when the cells were exposed to oxaliplatin, a two-fold induction of the *ERCC1* transcript was observed (Figure 2A). This stimulation of *ERCC1* expression was confirmed at the protein level by western blotting analysis (Figure 2B). Interestingly, we found that the combined oxaliplatin/cetuximab treatment induced a clear and strong reduction of *ERCC1* expression both at the mRNA and protein levels in the responding HCT-8 cells (Figure 2A and B) and not in the non-responsive HCT-116 cells. These results support the fact that cetuximab treatment in HCT-8 cells could elicit a loss of ERCC1 activity and prevent excision of platinum adducts from DNA, a process that may account for the responsiveness of this cell line.

### Oxaliplatin/cetuximab combination induced a dramatic decrease in cells in the S phase and enrichment in the G1 and G2/M phases in HCT-8 cells

**Figure 3:**
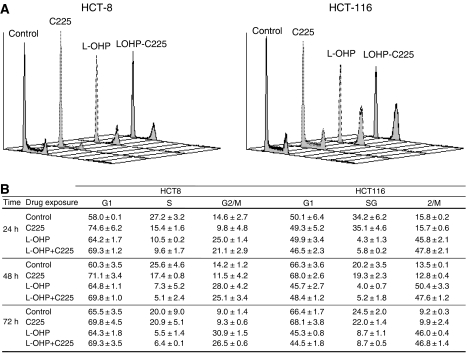
Effect of cetuximab, oxaliplatin, or combination exposure on cell cycle repartition of HCT-8 and HCT-116 cell lines. The cell cycle distribution of HCT-8 and HCT-116 cells was determined after (**A**) 24 h exposure to drug-free medium (
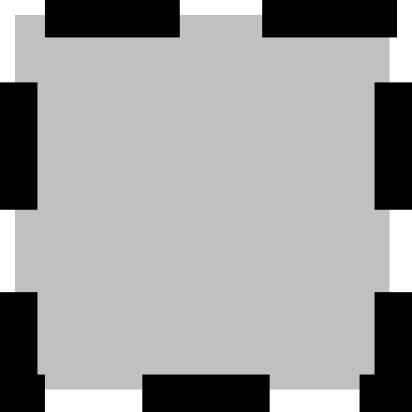
), 20 *μ*g ml^−1^ cetuximab (C225, 
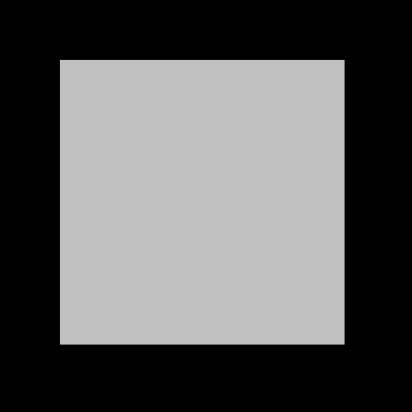
), 5 *μ*M oxaliplatin alone (L-OHP, 
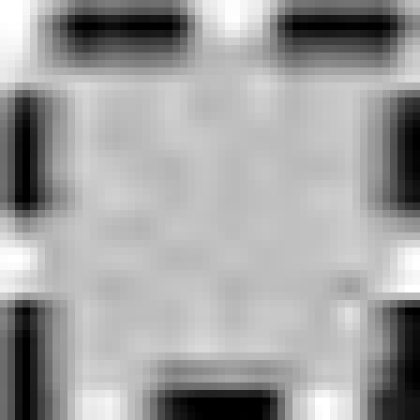
), or a combination of both (
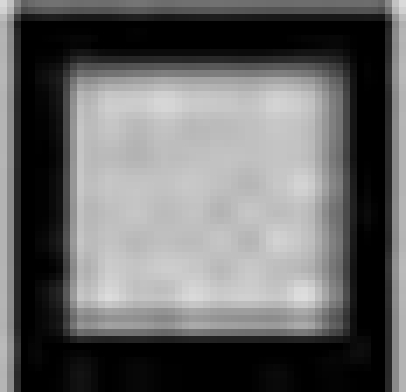
). (A representative experiment of three independent assessments is shown), and (**B**) kinetic evolution until 72 h of the percent of cells in G0/G1, S, and G2/M phases after exposure to either vehicle (control), 20 *μ*g ml^−1^ cetuximab daily (C225), 5 *μ*M oxaliplatin for 24 h followed by drug-free medium (L-OHP), or the combined treatment of oxaliplatin plus cetuximab for 24 h followed by 20 *μ*g ml^−1^ cetuximab daily (L-OHP+C225). Results are expressed as the mean±s.e.m. of three independent experiments.

To evaluate the portion of cells engaged in DNA processing at exposure, the effects on cell cycle distribution of each drug alone and combined were assessed in both the HCT-8 and HCT-116 cell lines (Figure 3). The 24-h exposure of HCT-8 cells to cetuximab or oxaliplatin alone induced a significant increase in cells in the G0/G1 phase from 57.9±0.1% for the untreated condition to 74.6±6.2 and 64.2±1.7%, respectively, when cells were treated with cetuximab or oxaliplatin. Moreover, oxaliplatin also induced an accumulation of cells in the G2/M phase (25.0±1.4 *vs* 14.6±2.7% for untreated cells). The combination of cetuximab with oxaliplatin resulted in the manifestation of both effects, an increase in the number of the cells in G0/G1 and G2/M phases (69.3±1.2 and 21.1±2.9%, respectively, *vs* 57.9±0.1 and 14.6±2.7% for the untreated condition) with a concomitant decrease in the number of cells in the S phase (9.6±1.7 *vs* 27.2±3.2% for untreated cells). Furthermore, kinetics evaluation of cell cycle progression until 72 h (Figure 3B) showed that oxaliplatin exposure led to the maintenance of the blockade through the G0/G1 and G2/M phases (64.3±1.8 and 30.9±1.5% *vs* 58.0±0.1 and 14.6±2.7% for untreated cells at 24 h) associated with a strong decrease in the number of cells in the S phase (5.5±1.4 *vs* 27.2±3.2% for control cells at 24 h). This cell cycle modification persisted to the 72-h time point.

In the HCT-116 cell line, oxaliplatin exposure induced an arrest in the G2/M phase from 24 h (45.8±2.1 *vs* 15.8±0.2% for untreated cells) to 72 h (46.0±0.4 *vs* 9.2±0.3% for untreated cells) resulting in a strong decrease in the number of cells in the S phase up to 72 h (8.7±0.5 *vs* 24.5±2.0% for control cells). Cetuximab alone had no effect; consequently, the effect from the combination of both drugs was assumed to be an effect of oxaliplatin alone.

### Oxaliplatin/cetuximab combination promotes an early apoptotic effect and inhibits survival through downregulation of AKT activation in HCT-8 cells

**Figure 4:**
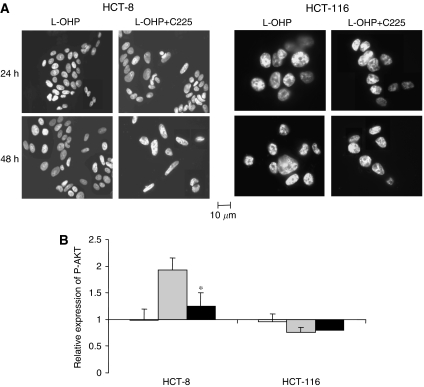
Proapoptotic effect of the oxaliplatin/cetuximab combination. (**A**) HCT-8 and HCT-116 cells were exposed to 5 *μ*M oxaliplatin (L-OHP) or 5 *μ*M oxaliplatin plus 20 *μ*g ml^−1^ cetuximab (L-OHP+C225) for 24 h. Cells were then stained with DAPI to visualise the nuclear morphology and to detect any pattern of chromatin condensation and nuclear DNA fragmentation that characterises apoptotic cells (a representative experiment of three independent assessments is shown). (**B**) Quantitation of phosphorylated AKT variations in HCT-8 and HCT-116 cells after 24 h exposure to 20 *μ*g ml^−1^ cetuximab (□), 5 *μ*M oxaliplatin (
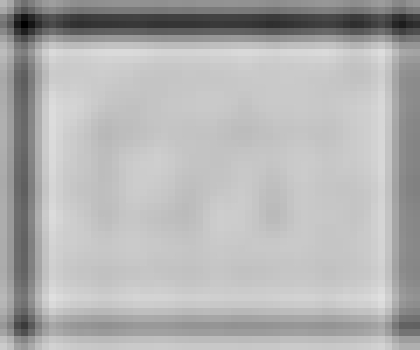
), or a combination of both (▪) compared to untreated cells. Results are expressed as the mean±s.e.m. of three independent experiments. ^*^P<0.05. Student's *t*-test compared to the effect of oxaliplatin alone.

We next studied the putative effect of cetuximab and/or oxaliplatin exposure on the morphology of cellular nuclei through detection of an apoptotic pattern of chromatin condensation and nuclear DNA fragmentation using DAPI fluorescent staining (Figure 4A). No significant apoptotic effect of cetuximab alone was observed for any cell line or treatment. HCT-8 cells treated with oxaliplatin displayed at *t*=48 h typical morphological features of apoptotic cells with condensed and fragmented nuclei. When oxaliplatin was combined with cetuximab, these features were observed at *t*=24 h, suggesting that the proapoptotic effect of oxaliplatin occurs 24 h earlier when combined with cetuximab. No differences were observed between oxaliplatin alone and combined with cetuximab in the HCT-116 cells. Obviously, for both cell lines, the cytotoxic effect of oxaliplatin was still present; this was characterised from the first 24 h and during the time course treatment by a decrease in the cell number, as visualised by fluorescent microscopy.

Because the PI3K/AKT pathway is a key point in the regulation of the balance between survival and apoptosis downstream of the signal transduction pathway regulated by EGFR, we performed quantification of the phosphorylated form of AKT to assess survival regulation in the cell lines after exposure to cetuximab and/or oxaliplatin (Figure 4B). In HCT-8 cells, oxaliplatin induced a significant induction of phospho-AKT levels by 1.9±0.2 (relative expression), which was significantly inhibited by the simultaneous exposure to cetuximab (*P*<0.05). On the other hand, in the HCT-116 cell line, we observed no difference between the effect on the phospho-AKT level of oxaliplatin alone or combined with cetuximab.

### Cetuximab enhances oxaliplatin-induced downregulation of multiple targets involved in DNA replication, recombination, and repair

**Figure 5:**
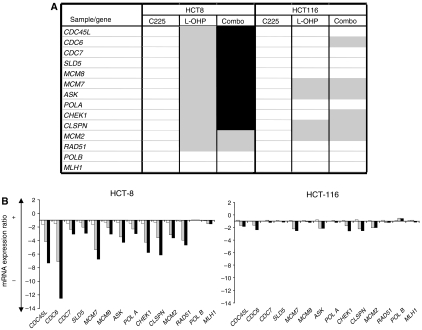
Multigene analysis of cetuximab, oxaliplatin, or a combination of both on expression of multiple genes involved in DNA replication, repair, and recombination. (**A**) Reverse transcription quantitative PCR analysis was performed on various genes involved in DNA repair, replication, and recombination in HCT-8 and HCT-116 cells exposed to either 20 *μ*g ml^−1^ cetuximab (C225), 5 *μ*M oxaliplatin (L-OHP), or a combination of both (Combo) for 24 h. Gene expression variation factors <2 (
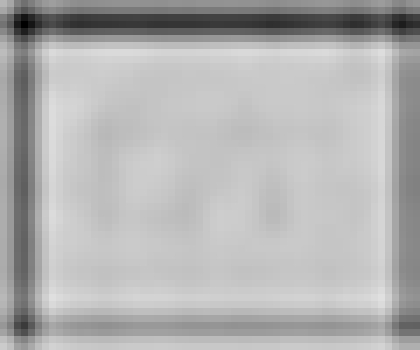
) and increased effect of oxaliplatin by more than 30% (▪) when combined with cetuximab are represented. (**B**) Focus on relative expression of genes involved in the initiation of DNA replication after 24 h exposure to 20 *μ*g ml^−1^ cetuximab (□), 5 *μ*M oxaliplatin (
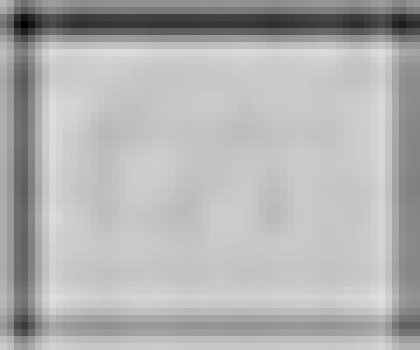
), or a combination of both (▪). To normalise gene expression between treated and control samples, control genes of *GAPDH* and *HPRT* were used. Relative quantitation of mRNA expression of each gene was performed in triplicate.

In addition to the NER pathway, many other potential DNA interactions manage resistance to platinum damage. These mechanisms include regulation of DNA replication by specialised DNA polymerases, mismatch repair, or recombination repair. Therefore, we performed a large-scale RT-QPCR analysis using low-density array technology to compare the transcriptional response between HCT-116 and HCT-8 cells after drug exposure. We assessed expression of multiple target genes involved in these adaptive or repair pathways as well as genes regulating the S-phase progression during the cell cycle (Figure 5). For each cell line, results are presented as the mRNA expression ratio between treated and untreated conditions. Results highlight very different profiles of response to oxaliplatin exposure between HCT-116 and HCT-8 cells (Figure 5), but cetuximab alone had no effect in these cell lines. Except for *PolB* and human *MLH1* genes, which were not significantly modulated in the two cell lines, oxaliplatin induced a more important downregulation of the replication and repair factors in HCT-8 than in HCT-116. The combination of the two treatments in HCT-8 tended to increase the downregulation of targets, and the effect was particularly obvious regarding *Claspin*, *CDC45*, and *CDC6* expressions, three major proteins involved in DNA replication initiation.

*CDC45L*, *CDC6*, *CDC7*, *SLD5*, *MCM7*, *ASK*, *polA*, and *MCM2* are all implicated in the initiation of DNA replication and their downregulation might be reflected in the blockade in the G1 phase of the cell cycle observed in the previously described experimental results.

## DISCUSSION

We previously demonstrated that cetuximab sensitised some colorectal cancer cell lines to the platinum-derived drug oxaliplatin (Balin-Gauthier *et al*, 2006). In the present study, we used the HCT-8 and HCT-116 cell lines to investigate the cellular and molecular mechanisms and pathways involved in the synergistic response to oxaliplatin/cetuximab combination observed in the HCT-8 cell line compared to the non-responsive HCT-116 cell line.

The HCT-8 and HCT 116 cell lines are well characterised in their mutation status of *K-RAS*, *B-RAF*, and *EGFR* and alteration of *EGFR* gene copy number (CISH study, data not shown), implicated with anti-EGFR mAbs response. HCT-8 cells have a wild-type status concerning *K-RAS*, *B-RAF*, and *EGFR* genes and are not amplified for *EGFR* (Balin-Gauthier *et al*, 2006; Benvenuti *et al*, 2007), whereas HCT-116 cells are *K-RAS* mutated, *B-RAF* wild type and *EGFR* wild type and non-amplified (Balin-Gauthier *et al*, 2006; Benvenuti *et al*, 2007).

In the responsive HCT-8 colorectal cancer cell line, cetuximab induces the stimulation of platinum–DNA adduct formation. This upregulation is associated with reduced expression of the NER key factor ERCC1, both at the mRNA and protein levels. Moreover, we observed a reduced expression of factors involved in DNA replication initiation, which correlates with an enrichment of cells in the G1 phase of the cell cycle plus the stimulation of the apoptosis pathway outcome with an acceleration of apoptosis. None of these changes occurred in the non-responsive HCT-116 cell line, pointing out multiple interactions between oxaliplatin pharmacology and the EGFR signalling transduction pathway. Finally, the analysis of the gene expression modulation highlights the inhibition of DNA replication initiation as a signature of oxaliplatin/cetuximab synergistic interaction.

When a synergistic interaction occurred between oxaliplatin and cetuximab in HCT-8 cells, platinum–DNA adduct formation increased by 20%, suggesting two different but possibly linked mechanisms. What was unknown was whether cetuximab inhibits the oxaliplatin detoxification pathway and/or cetuximab inhibits the DNA–platinum adduct repair pathway. Thus, we studied these two possibilities. Because the major system of oxaliplatin detoxification uses the GSH cycle through activation of GSTs (Pendyala and Creaven, 1993), we investigated the effect of drug exposure on GST activity. Because any drug alone or in combination did not induce any modulation of GST activity, we focused on oxaliplatin–DNA adduct repair whose main cellular process is the NER.

When HCT-8 cells were exposed to a strong dose of oxaliplatin in combination with cetuximab, the level of adduct stabilised 24-h after exposure termination, but it continuously decreased under exposure to oxaliplatin alone. Moreover, these results are consistent with those of Xu *et al* (2003), demonstrating that gefitinib, a tyrosine kinase inhibitor of EGFR, modestly enhanced cellular oxaliplatin accumulation and platinum–DNA adduct levels and significantly inhibited removal of Platinum–DNA adducts. Taken together, our results and those of previously published works suggest that inhibition of the EGFR pathway may stabilise platinum–DNA adduct content through inhibition of platinum–DNA adduct repair.

Nucleotide excision repair is the main DNA repair mechanism involved in oxaliplatin–DNA adduct repair (Reed, 1998; Chaney *et al*, 2005). This mechanism is associated with adduct recognition proteins such as XPA and XPC (*xeroderma pigmentosum A and C:* DNA-lesion recognition proteins) and with proteins such as ERCC1, which catalyses excision of the damaged nucleotide. Because ERCC1 catalyses the 5′ incision at the damage site (Reed, 1998), its expression and functionality are crucial for NER activity. Moreover, its overexpression has been reported to be correlated with oxaliplatin resistance (Arnould *et al*, 2003). Thus, we investigated whether the NER could be targeted by the combined treatment of oxaliplatin plus cetuximab through the regulation of *ERCC1* and *XPA* expression at the translational and transcriptional levels.

Unlike studies demonstrating that *ERCC1* basal level could predict the effect of oxaliplatin alone (Raymond *et al*, 2002), in our study, this level was similar between HCT-116 and HCT-8 cells (data not shown) and thus could not predict a synergistic interaction between oxaliplatin and cetuximab. However, we have shown that oxaliplatin induces a two-fold upregulation of *ERCC1* mRNA and protein expression in HCT-8 but not in HCT-116 cells. It has been reported that oxaliplatin induces *ERCC1* expression in cell lines that display resistance to platinum drugs (Youn *et al*, 2004). We demonstrated that the oxaliplatin/cetuximab combination inhibits the oxaliplatin-induced upregulation of *ERCC1* observed in HCT-8 cells. This inhibition of expression is observed both at the mRNA and protein levels, suggesting that the cetuximab/oxaliplatin combination inhibits *ERCC1* induction and consequently NER activity through this transcriptional regulation. These data also suggest that the sensitisation of HCT-8 cells to oxaliplatin under cetuximab exposure is partly the result of inhibition of the main mechanism implicated in the repair of platinum–DNA adducts. The EGFR pathway could control the regulation of *ERCC1* expression and thus oxaliplatin resistance. We know that the EGFR signalling pathway can regulate transcription through the activation of oncogene transcription factors such as *c-jun*, *c-myc*, and *c-fos* (Yarden, 2001). Interestingly, *ERCC1* gene transcription can be regulated by the AP-1 transcription factor (*c-Fos* and *c Jun* *dimer*) that binds to its promoter region (Youn *et al*, 2004), involving this factor in the mechanisms of oxaliplatin resistance.

Deficiency of DNA repair drives cells to undergo apoptosis, which led us to study the putative effect of cetuximab and/or oxaliplatin exposure on this specific cell death process. In HCT-8 cells exposed to oxaliplatin/cetuximab, morphologic changes associated with apoptosis (DNA condensation and fragmentation) occurred 24 h earlier than in cells exposed to oxaliplatin alone and could play a role in the enhanced antiproliferative effects that we observed previously (Chaney *et al*, 2005). In HCT-8 cells, oxaliplatin exposure promoted survival through AKT activation; cetuximab exposure concomitant with oxaliplatin inhibited this regulation and induced restoration of apoptosis through the inhibition of oxaliplatin-induced AKT activation. On the other hand, when the non-responsive HCT-116 cells were exposed to oxaliplatin either alone or combined with cetuximab, we observed similar nucleic morphologic changes. At the same time, no effect on AKT phosphorylation was observed in this cell line. Because activation of survival through the PI3K/AKT pathway is one mechanism by which cells can resist to cytotoxic drug exposure (Kim *et al*, 2005), such data suggest that the inhibition of AKT activation and related effects on apoptosis could be part of the HCT-8 synergistic response to the oxaliplatin/cetuximab combination. On the other hand, in HCT-116, a K-Ras mutated cell line, EGF-R inhibition had no effect either on the MAPK pathway (data not shown) or on AKT phosphorylation. This lack of effect could explain the lack of efficacy of cetuximab in K-Ras-mutated colorectal cancer (Benvenuti *et al*, 2007).

Experiments addressing cell cycle regulation showed that cetuximab treatment induced a weak (10%) enrichment in the G0/G1 phase only in the HCT-8 cell line. These results are consistent with those in the literature, including our previous work (Kiyota *et al*, 2002; Chaney *et al*, 2005), in which we reported that EGFR inhibitors are generally characterised by a cytostatic effect associated with enrichment of the G0/G1 phase of the cell cycle. Moreover, for each cell line, we showed that the effects on cell cycle of oxaliplatin/cetuximab together are additive of the effect of each molecule used alone.

Our results led us to extend our study to multiple gene analysis of targets implicated in DNA repair, replication, and recombination. The results of these studies suggested that under oxaliplatin treatment, the presence of the oxaliplatin adducts prevents the firing of new replication origins, explaining the low number of cells in the S phase. Such inhibition of licensing or replication initiation could prevent cells from duplicating long patches of DNA and consequently promote DNA translesion or homologous recombination repair to process DNA damage. In our models, cetuximab alone had no effect in downregulation mechanisms of replication initiation. On the other hand, when used in combination with oxaliplatin, it modified oxaliplatin adduct repair and induced modifications in the early steps of DNA replication in the HCT-8 cell line. To conclude, the present study shows for the first time that inhibition of the EGFR transduction pathway by cetuximab sensitises cells to oxaliplatin via the downregulation of mechanisms implicated in oxaliplatin resistance. The characteristics of the synergistic response to the oxaliplatin/cetuximab combination in the HCT-8 cell line led us to highlight the importance of DNA replication initiation as a critical step that could be a molecular signature of response to this combination.

## References

[bib1] Arnould S, Hennebelle I, Canal P, Bugat R, Guichard S (2003) Cellular determinants of oxaliplatin sensitivity in colon cancer cell lines. Eur J Cancer 39: 112–119 12504667 10.1016/s0959-8049(02)00411-2

[bib2] Balin-Gauthier D, Delord JP, Rochaix P, Mallard V, Thomas F, Hennebelle I, Bugat R, Canal P, Allal C (2006) *In vivo* and *in vitro* antitumor activity of oxaliplatin in combination with cetuximab in human colorectal tumor cell lines expressing different level of EGFR. Cancer Chemother Pharmacol 57: 709–718 16320055 10.1007/s00280-005-0123-3

[bib3] Benvenuti S, Sartore-Bianchi A, Di Nicolantonio F, Zanon C, Moroni M, Veronese S, Siena S, Bardelli A (2007) Oncogenic activation of the RAS/RAF signaling pathway impairs the response of metastatic colorectal cancers to anti-epidermal growth factor receptor antibody therapies. Cancer Res 67(6): 2643–2648 17363584 10.1158/0008-5472.CAN-06-4158

[bib4] Blyumenberg G, Gorbacheva LB, Gorbunova VA, Kozachenko VP, Lankin VZ (1996) Factors of the ovarian cancer resistance to combined chemotherapy with platinum preparations. Bull Exp Biol Med 122(6): 1213–1216 9280465

[bib5] Carpenter G, Cohen S (1990) Epidermal growth factor. J Biol Chem 265: 7709–7712 2186024

[bib6] Chaney SG, Campbell SL, Bassett E, Wu Y (2005) Recognition and processing of cisplatin– and oxaliplatin–DNA adducts. Crit Rev Oncol Hematol 53: 3–11 15607931 10.1016/j.critrevonc.2004.08.008

[bib7] Cunningham D, Humblet Y, Siena S, Khayat D, Bleiberg H, Santoro A, Bets D, Mueser M, Harstrick A, Verslype C, Chau I, Van Cutsem E (2004) Cetuximab monotherapy and cetuximab plus irinotecan in irinotecan-refractory metastatic colorectal cancer. N Engl J Med 351: 337–345 15269313 10.1056/NEJMoa033025

[bib8] Habig WH, Pabst MJ, Jakoby WB (1974) Glutathione *S*-transferases. The first enzymatic step in mercapturic acid formation. J Biol Chem 249: 7130–7139 4436300

[bib9] Kim D, Cheng GZ, Lindsley CW, Yang H, Cheng JQ (2005) Targeting the phosphatidylinositol-3 kinase/Akt pathway for the treatment of cancer. Curr Opin Investig Drugs 6: 1250–1258 16370391

[bib10] Kiyota A, Shintani S, Mihara M, Nakahara Y, Ueyama Y, Matsumura T, Tachikawa T, Wong DT (2002) Anti-epidermal growth factor receptor monoclonal antibody 225 upregulates p27(KIP1) and p15(INK4B) and induces G1 arrest in oral squamous carcinoma cell lines. Oncology 63: 92–98 12187077 10.1159/000065726

[bib11] Lee JC, Wang ST, Chow NH, Yang HB (2002) Investigation of the prognostic value of coexpressed erbB family members for the survival of colorectal cancer patients after curative surgery. Eur J Cancer 38: 1065–1071 12008194 10.1016/s0959-8049(02)00004-7

[bib12] Pendyala L, Creaven PJ (1993) *In vitro* cytotoxicity, protein binding, red blood cell partitioning, and biotransformation of oxaliplatin. Cancer Res 53: 5970–5976 8261411

[bib13] Porebska I, Harlozinska A, Bojarowski T (2000) Expression of the tyrosine kinase activity growth factor receptors (EGFR, ERBB2, ERBB3) in colorectal adenocarcinomas and adenomas. Tumor Biol 21: 105–115 10.1159/000030116 10686540

[bib14] Prewett MC, Hooper AT, Bassi R, Ellis LM, Waksal HW, Hicklin DJ (2002) Enhanced antitumor activity of anti-epi-dermal growth factor receptor monoclonal antibody IMC-C225 in combination with irinotecan (CPT-11) against human colorectal tumor xenografts. Clin Cancer Res 8: 994–1003 12006511

[bib15] Raymond E, Faivre S, Chaney S, Woynarowski J, Cvitkovic E (2002) Cellular and molecular pharmacology of oxaliplatin. Mol Cancer Ther 1: 227–235 12467217

[bib16] Reed E (1998) Platinum-DNA adduct, nucleotide excision repair and platinum based anti-cancer chemotherapy. Cancer Treat Rev 24: 331–344 9861196 10.1016/s0305-7372(98)90056-1

[bib17] Saltz LB, Meropol NJ, Loehrer Sr PJ, Needle MN, Kopit J, Mayer RJ (2004) Phase II trial of cetuximab in patients with refractory colorectal cancer that expresses the epidermal growth factor receptor. J Clin Oncol 22: 1201–1208 14993230 10.1200/JCO.2004.10.182

[bib18] Vandesompele J, De Preter K, Pattyn F, Poppe B, Van Roy N, De Paepe A, Speleman F (2002) Accurate normalization of real-time quantitative RT-PCR data by geometric averaging of multiple internal control genes. Genome Biol 3: RESEARCH0034 12184808 10.1186/gb-2002-3-7-research0034 PMC126239

[bib19] Wernyj RP, Morin PJ (2004) Molecular mechanisms of platinum resistance: still searching for the Achilles’ heel. Drug Resist Updat 7(4–5): 227–232 15533760 10.1016/j.drup.2004.08.002

[bib20] Xu JM, Azzariti A, Severino M, Lu B, Colucci G, Paradiso A (2003) Characterization of sequence-dependent synergy between ZD1839 (‘Iressa’) and oxaliplatin. Biochem Pharmacol 66: 551–563 12906920 10.1016/s0006-2952(03)00291-0

[bib21] Yarden Y (2001) The EGFR family and its ligands in human cancer. signalling mechanisms and therapeutic opportunities. Eur J Cancer 37(Suppl 4): S3–S8 10.1016/s0959-8049(01)00230-1 11597398

[bib22] Youn CK, Kim MH, Cho HJ, Kim HB, Chang IY, Chung MH, You HJ (2004) Oncogenic H-Ras up-regulates expression of ERCC1 to protect cells from platinum-based anticancer agents. Cancer Res 64: 4849–4857 15256455 10.1158/0008-5472.CAN-04-0348

[bib23] Yu JJ, Mu C, Dabholkar M, Bostick-Bruton F, Reed E (1998) Alternative splicing of ERCC1 and cisplatin–DNA adduct repair in human tumor cell lines. Int J Mol Med 1: 617–620 9852275 10.3892/ijmm.1.3.617

